# Monocytic myeloid-derived suppressor cells as an immune indicator of early diagnosis and prognosis in patients with sepsis

**DOI:** 10.1186/s12879-024-09290-4

**Published:** 2024-04-13

**Authors:** Juanjuan Cui, Wen Cai, Jing Lin, Li Zhang, Youhan Miao, Ying Xu, Weifeng Zhao

**Affiliations:** 1 Department of Infectious Diseases, The First Affiliated Hospital of Soochow University, No. 899 Pinghai Road, Gusu District, 215006 Suzhou, China; 2 Center of Clinical Laboratory, The First Affiliated Hospital of Soochow University, No. 899 Pinghai Road, Gusu District, 215006 Suzhou, China; 3 Department of Infectious Diseases, The Third Affiliated Hospital of Nantong University, No. 60 Qingnian Middle Road, Chongchuan District, 226006 Nantong, China

**Keywords:** Low-density neutrophils (LDNs), Monocytic myeloid-derived suppressor cells (M-MDSCs), Polymorphonuclear myeloid-derived suppressor cells (PMN-MDSCs), Sepsis, Infection, Diagnosis, Prognosis

## Abstract

**Background:**

Immunosuppression is a leading cause of septic death. Therefore, it is necessary to search for biomarkers that can evaluate the immune status of patients with sepsis. We assessed the diagnostic and prognostic value of low-density neutrophils (LDNs) and myeloid-derived suppressor cells (MDSCs) subsets in the peripheral blood mononuclear cells (PBMCs) of patients with sepsis.

**Methods:**

LDNs and MDSC subsets were compared among 52 inpatients with sepsis, 33 inpatients with infection, and 32 healthy controls to investigate their potential as immune indicators of sepsis. The percentages of LDNs, monocytic MDSCs (M-MDSCs), and polymorphonuclear MDSCs (PMN-MDSCs) in PBMCs were analyzed. Sequential organ failure assessment (SOFA) scores, C-reactive protein (CRP), and procalcitonin (PCT) levels were measured concurrently.

**Results:**

The percentages of LDNs and MDSC subsets were significantly increased in infection and sepsis as compared to control. MDSCs performed similarly to CRP and PCT in diagnosing infection or sepsis. LDNs and MDSC subsets positively correlated with PCT and CRP levels and showed an upward trend with the number of dysfunctional organs and SOFA score. Non-survivors had elevated M-MDSCs compared with that of patients who survived sepsis within 28 days after enrollment.

**Conclusions:**

MDSCs show potential as a diagnostic biomarker comparable to CRP and PCT, in infection and sepsis, even in distinguishing sepsis from infection. M-MDSCs show potential as a prognostic biomarker of sepsis and may be useful to predict 28-day hospital mortality in patients with sepsis.

**Supplementary Information:**

The online version contains supplementary material available at 10.1186/s12879-024-09290-4.

## Introduction

Sepsis is a dysregulated host response to infection that affects millions of people worldwide each year [1]. Its mortality rate ranges from 16.67 to 33.33% [2]. Timely assessment and appropriate treatment during the initial hours of sepsis can improve outcomes [3]. The leading cause of sepsis-related deaths between 1990 and 2017 was infection [4]. Conditions like cytokine storms and sepsis-induced immunosuppression increase sepsis severity and induce poor prognosis [5]. Monitoring the immune status of patients with sepsis and providing immunomodulatory treatment may improve survival rates [6]. Some biomarkers have been reported to predict sepsis in infection; however, the identification of further suitable immune indicators to assess the host immune status is vital for early diagnosis and better prognostic power.

Low-density neutrophils (LDNs) are found in peripheral blood mononuclear cells (PBMCs) after density gradient centrifugation of whole blood [7]. Depending on their function, LDNs are characterized as immunosuppressive, proinflammatory, or immature neutrophils^[7]^. LDN counts are typically small in healthy donors [8], but are elevated in alcohol-associated hepatitis [9], *Mycobacterium tuberculosis* infection [10], systemic lupus erythematosus (SLE) [11], severe obesity [12], myocardial infarction [13] and abdominal surgery [14]. Myeloid-derived suppressor cells (MDSCs) are pathologically activated neutrophils and monocytes with immunosuppressive activities [15]. Based on their origin, MDSCs are classified into two major groups: granulocytic/polymorphonuclear MDSCs (PMN-MDSCs) and monocytic MDSCs (M-MDSCs) [15]. M-MDSCs are defined as CD11b^+^CD14^+^HLA-DR^−/lo^CD15^−^ cells and PMN-MDSCs as CD11b^+^CD14^−^CD15^+^ cells in human PBMCs [16]. MDSCs, which are widely recognized for their ability to attenuate immune surveillance and their antitumor properties, play a role in deleterious immunosuppressive processes.

Immunotherapy can normalize immune cells and improve the prognosis of patients. Thus, we explored the possibility of using immune cells as biomarkers for sepsis. We assessed whether the levels of immunosuppressive indicators, LDNs and MDSC subsets in PBMCs, differ in patients with infection or sepsis. In addition, we evaluated the association between immune cells and the severity of sepsis to find a potential prognostic biomarker of sepsis.

## Materials and methods

### Study design and participants

**Fig. 1:**
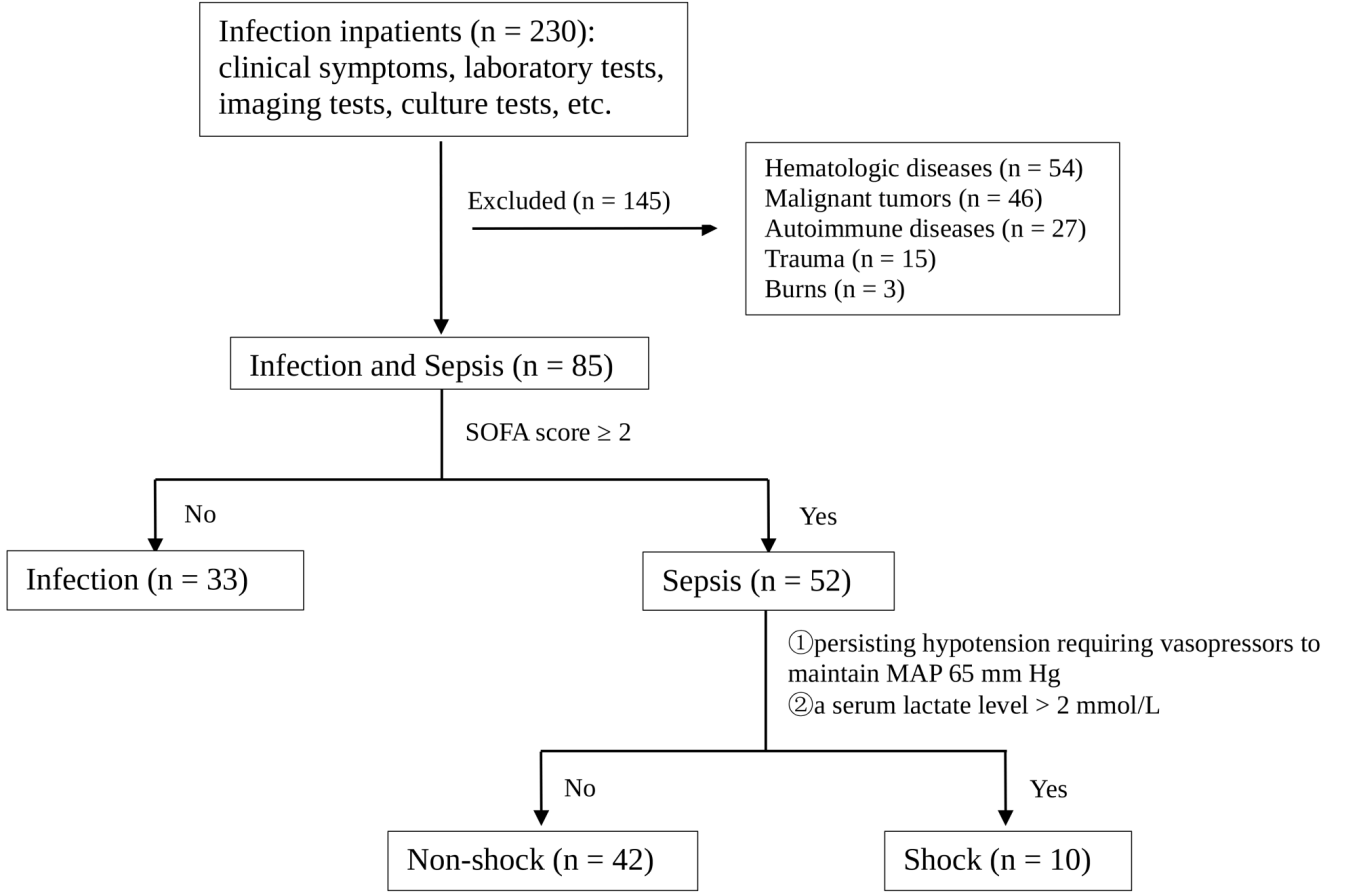
The study flow of patient selection and diagnosis

This prospective study analyzed a total of 85 inpatients, including 52 patients diagnosed with sepsis according to “The Third International Consensus Definitions for Sepsis and Septic Shock” (Sepsis-3) [1], 33 patients diagnosed with infection, and 32 healthy controls at the First Affiliated Hospital of Soochow University from December 2021 to April 2023. The exclusion criteria were pregnancy; age < 18 years; received immunomodulatory medications and the presence of hematologic diseases, malignant tumors, autoimmune diseases, trauma, or burns (Fig. 1). Furthermore, subgroups were used to categorize infection and sepsis groups based on infection sites and pathogenic pathogens. LDNs, M-MDSCs, and PMN-MDSCs in PBMCs were detected using flow cytometry at the time of inclusion. Cell counts of leukocytes, lymphocytes, monocytes, and neutrophils; percentages of lymphocytes, monocytes, and neutrophils; and reported infection biomarkers, including procalcitonin (PCT) and C-reactive protein (CRP), were collected. The monocyte-to-lymphocyte ratio (MLR), neutrophil-to-lymphocyte ratio (NLR), and sequential organ failure assessment (SOFA) scores were calculated at admission. PCT levels < 0.02 ng/mL were rounded to 0.02 ng/mL. We compiled clinical characteristics and laboratory results within 48 h confirmed sepsis or infection, and collected Ethylenediamine tetraacetic acid (EDTA) anti-coagulant whole blood at the same time.

### Protocol of LDNs and MDSC subsets in PBMCs

PBMCs were isolated from EDTA anti-coagulant whole blood using Lymphoprep™ (STEMCELL, Vancouver, BC, Canada) according to the manufacturer’s instructions. PBMCs were washed once with phosphate-buffered saline (PBS). The following antibodies (all obtained from Bearl, Beckman Coulter, Miami, FL, USA) were added to the PBMCs: anti-human CD11b-PC7 (clone number: Bear1), anti-human HLA-DR-FITC (clone number: Bear1), anti-human CD14-APC (clone number: RMO52), and anti-human CD15-PE (clone number: 80H5), after which the solution was incubated in darkness for 20 min. RBC lysis solution (Qiagen, Hilden, NRW, Germany) was added, after which the solution was incubated for another 10 min. The PBMCs were rinsed once with PBS. At least 50,000 cells from each sample were analyzed using Navios (Beckman Coulter, Brea, CA, USA). The results were expressed as the percentages of cells. Flow cytometry data were analyzed using Kaluza software (Beckman Coulter, Brea, CA, USA). The gating strategy was shown in Fig. S1.

### Statistical analysis

Categorical variables were presented as numbers (%) and compared using the chi-square test or Fisher’s exact test. Continuous variables were presented as medians (interquartile ranges) and compared using the Mann–Whitney *U* test or Kruskal–Wallis *H* test. The nonparametric Spearman’s correlation was used to calculate the coefficients. The diagnostic and prognostic performances of the biomarkers were assessed by drawing receiver operating characteristic (ROC) curves and calculating the area under the curve (AUC). DeLong’s test was used to determine whether there were significant differences between the immune indicators and CRP or PCT by comparing the AUCs. Ordinal logistic regression analysis was used to identify biomarkers associated with the risk of sepsis as assessed using odds ratios (OR). The Jonckheere–Tepstra trend test was used to verify whether LDNs and MDSC subsets increased with sepsis severity. The Kaplan–Meier survival curve was plotted to show the 28-day survival rate of patients with sepsis and the log-rank (Mantel–Cox) test was used to compare survival curves. Univariate Cox regression analysis was used to identify prognostic biomarkers associated with sepsis in 28-day mortality and then assessed using hazard ratios (HR). All tests were two-tailed and *P* values below 0.05 were considered significant. Statistical analyses were conducted using GraphPad Prism 8.0 (San Diego, CA, USA) or SPSS software (version 24.0, Chicago, Illinois, USA).

## Results

### Participant characteristics

The clinical characteristics of healthy controls, infection, and sepsis are shown in Table 1. There were no notable differences in gender or age among the groups. The number of dysfunctional organs and SOFA scores varied significantly among the three groups and between the infection group and sepsis group. Statistical differences were observed in the cell counts and percentages of lymphocytes, and neutrophils, as well as in NLR and MLR, not only among the three groups but also between infection and sepsis. There was no difference in monocyte count between infection and sepsis.

**Table 1:** Baseline characteristics of patients

	Healthy Controls (*n* = 32)	Infection (*n* = 33)	Sepsis (*n* = 52)	*P* value^*^	*P* value^#^
**Demographics and clinical characteristics**					
Male, n (%)	16 (50.0)	18 (54.5)	36 (69.2)	0.167	0.170
Age (year)	60.00 (53.25–65.50)	63.00 (34.00–73.50)	66.50 (53.00–74.75)	0.076	0.176
Hypertension, n (%)	0 (0.0)	9 (27.3)	24 (46.2)	< 0.001	0.082
Diabetes, n (%)	0 (0.0)	11 (33.3)	20 (38.5)	< 0.001	0.632
**Organ dysfunction, n (%)**					
Respiratory	0 (0.0)	0 (0.0)	28 (53.8)	< 0.001	< 0.001
Cardiovascular	0 (0.0)	5 (15.2)	40 (76.9)	< 0.001	< 0.001
Hepatic	0 (0.0)	3 (9.4)	30 (57.7)	< 0.001	< 0.001
Hematologic	0 (0.0)	0 (0.00)	25 (48.1)	< 0.001	< 0.001
Neurologic	0 (0.0)	0 (0.00)	28 (53.8)	< 0.001	< 0.001
Renal	0 (0.0)	0 (0.00)	18 (34.6)	< 0.001	0.006
**Severity of infection**					
Number of dysfunctional organs	0 (0–0)	0 (0–0.5)	3.00 (1.25–5.00)	< 0.001	< 0.001
SOFA score	0 (0–0)	0 (0–0.5)	7 (3–12)	< 0.001	< 0.001
**Laboratory examinations**					
Leukocyte count (×10^9^/L)	5.26 (4.34–6.70)	7.66 (5.59–10.48)	11.02 (7.12–15.82)	< 0.001	0.005
Lymphocyte count (×10^9^/L)	1.72 (1.38–2.08)	1.35 (0.90–1.97)	0.76 (0.45–1.26)	< 0.001	< 0.001
Monocyte count (×10^9^/L)	0.35 (0.24–0.40)	0.50 (0.35–0.72)	0.50 (0.27–0.83)	< 0.001	0.917
Neutrophil count (×10^9^/L)	2.92 (2.31–4.30)	5.42 (3.35–8.05)	9.63 (5.67–14.23)	< 0.001	< 0.001
Lymphocyte percentage (%)	32.10 (26.45–38.49)	16.50 (12.80–28.20)	6.55 (3.95–10.88)	< 0.001	< 0.001
Monocyte percentage (%)	5.60 (4.95–7.48)	7.00 (4.65–8.75)	4.35 (2.33–6.83)	0.003	0.003
Neutrophil percentage (%)	59.35 (53.48–64.45)	73.00 (60.45–78.00)	88.15 (79.20–91.58)	< 0.001	< 0.001
MLR	0.189 (0.148–0.235)	0.390 (0.228–0.664)	0.598 (0.306–0.984)	< 0.001	0.025
NLR	1.853 (1.410–2.437)	4.361 (2.190–6.357)	13.03 (7.660–23.35)	< 0.001	< 0.001

LDNs and MDSC subsets as diagnostic biomarkers of sepsis or infection

*Abbreviations*: SOFA, sequential organ failure assessment; MLR, monocyte-to-lymphocyte ratio; NLR, neutrophil-to-lymphocyte ratio

^*^Comparison among the three groups

^#^Comparison between infection group and sepsis group

### LDNs and MDSC subsets as diagnostic biomarkers of sepsis or infection

**Fig. 2:**
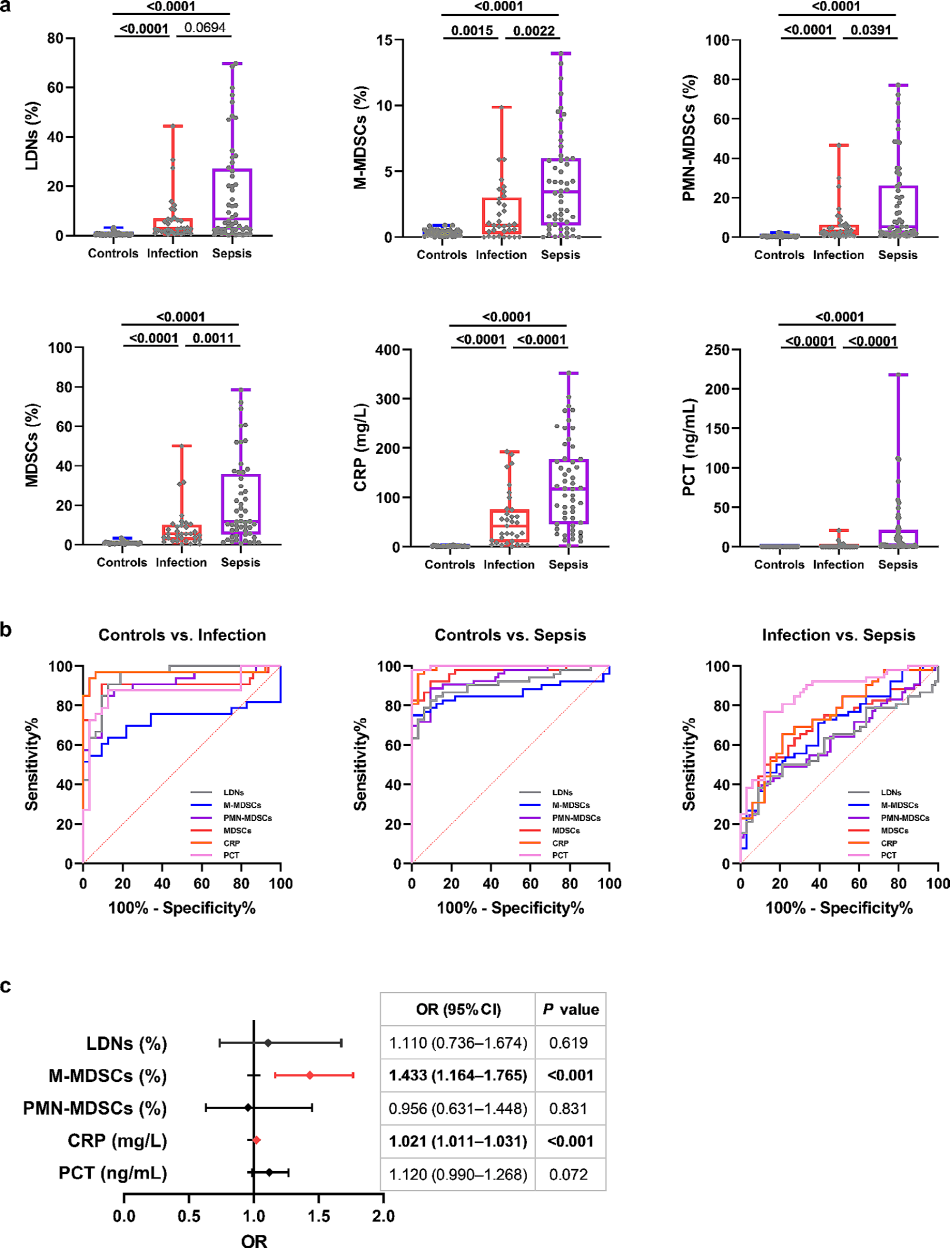
The diagnostic value of LDNs and MDSC subsets in sepsis. LDNs, MDSC subsets, CRP, and PCT levels in patients with healthy controls, infection, and sepsis (**a**). ROC curves for biomarkers to diagnose and distinguish between sepsis and infection (**b**). Forest plot showing the results of ordinal logistic regression analysis (**c**). OR and CIs significantly associated with sepsis were shown in red when OR *>* 1 and in blue when OR *<* 1. *Abbreviations:* ROC, receiver operator characteristics; OR, odds ratio; CI, confidence interval; LDNs, low-density neutrophils; MDSCs, myeloid-derived suppressor cells; M-MDSCs, monocytic MDSCs; PMN-MDSCs, polymorphonuclear MDSCs; CRP, C-reactive protein; PCT, procalcitonin

**Fig. 3:**
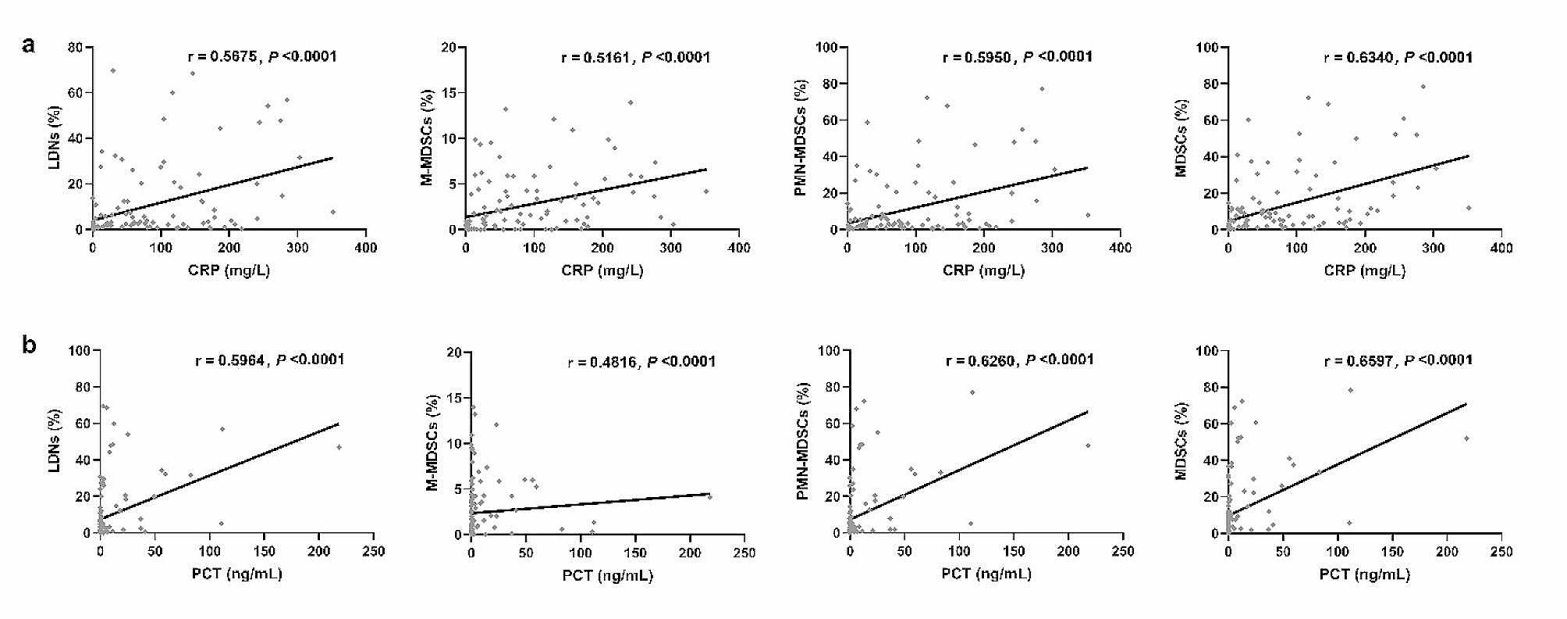
The correlations between immune indicators with CRP (**a**) or PCT (**b**). *Abbreviations:* LDNs, low-density neutrophils; MDSCs, myeloid-derived suppressor cells; M-MDSCs, monocytic MDSCs; PMN-MDSCs, polymorphonuclear MDSCs; CRP, C-reactive protein; PCT, procalcitonin

Typical flow diagrams of LDNs, M-MDSCs, and PMN-MDSCs in each group are shown (Fig. S2). The sepsis group had the highest percentages of LDNs and MDSC subsets, followed by the infection group, and the healthy controls had the lowest percentages (Fig. 2a). LDNs, PMN-MDSCs, MDSCs, and CRP were better diagnostic markers of infection than M-MDSCs and PCT. MDSCs, CRP, and PCT showed higher diagnostic values for sepsis than LDNs, M-MDSCs, and PMN-MDSCs. PCT was the best biomarker for differentially diagnosing infection and sepsis, with an AUC of 0.851 (0.765–0.936), outperforming LDNs, M-MDSCs, PMN-MDSCs, and MDSCs (Fig. 2b; Table 2). Ordinal logistic regression analysis showed that M-MDSCs increased the odds of sepsis by 1.433 (95% CI 1.164–1.765, *P* < 0.001). In addition, CRP (OR 1.021, 95% CI 1.011–1.031, *P* < 0.001) was a risk factor of sepsis (Fig. 2c). The percentages of LDNs, M-MDSCs, PMN-MDSCs, and MDSCs showed positive correlations with the levels of CRP and PCT (Fig. 3).

**Table 2:** Diagnostic efficacy of biomarkers for sepsis and infection

	AUC (95%CI)	Cutoff value	Sensitivity (%)	Specificity (%)		PPV (%)	NPV (%)	*P* value	*P* value (vs. CRP)	*P* value (vs. PCT)
**Healthy Controls vs. Infection**										
LDNs (%)	0.942 (0.888–0.996)	> 0.900	90.91	87.50		88.24	93.33	< 0.001	0.536	0.222
M-MDSCs (%)	0.726 (0.586–0.866)	> 0.913	51.52	100.0		100.0	68.09	0.002	**0.002**	0.076
PMN-MDSCs (%)	0.908 (0.833–0.983)	> 0.788	84.85	90.63		90.32	87.88	< 0.001	0.226	0.587
MDSCs (%)	0.904 (0.815–0.993)	> 1.454	90.91	90.63		90.91	93.55	< 0.001	0.247	0.658
CRP (mg/L)	0.967 (0.912–1.023)	> 3.565	93.94	96.88		96.88	96.88	< 0.001	-	**0.033**
PCT (ng/mL)	0.873 (0.776–0.970)	> 0.032	87.88	87.50		87.88	90.32	< 0.001	**0.033**	-
**Healthy Controls vs. Sepsis**										
LDNs (%)	0.908 (0.845–0.971)	> 1.085	82.69	90.63		93.48	74.36	< 0.001	**0.004**	**0.011**
M-MDSCs (%)	0.863 (0.779–0.947)	> 0.927	75.00	100.0		100.0	69.57	< 0.001	**0.002**	**0.003**
PMN-MDSCs (%)	0.938 (0.891–0.986)	> 0.834	90.38	90.63		94.00	82.86	< 0.001	**0.011**	**0.030**
MDSCs (%)	0.966 (0.930–1.001)	> 1.428	92.31	90.63		94.12	85.29	< 0.001	0.073	0.183
CRP (mg/L)	0.998 (0.994–1.002)	> 7.870	98.08	100.0		100.0	94.12	< 0.001	-	0.349
PCT (ng/mL)	0.992 (0.978–1.005)	> 0.071	96.15	96.88		98.04	91.18	< 0.001	0.349	-
**Infection vs. Sepsis**										
LDNs (%)	0.617 (0.499–0.736)	> 14.20	38.46	90.91		90.91	57.69	0.053	0.082	**0.001**
M-MDSCs (%)	0.696 (0.583–0.809)	> 3.962	46.15	87.88		82.14	49.12	0.001	0.466	**0.025**
PMN-MDSCs (%)	0.642 (0.525–0.759)	> 11.56	42.31	87.88		84.62	49.15	0.017	0.150	**0.002**
MDSCs (%)	0.708 (0.597–0.819)	> 11.28	53.85	84.85		84.85	53.85	< 0.001	0.567	**0.026**
CRP (mg/L)	0.749 (0.643–0.854)	> 78.21	65.38	78.79		85.37	61.36	< 0.001	-	**0.049**
PCT (ng/mL)	0.851 (0.765–0.936)	> 0.410	76.92	87.88		90.91	70.73	< 0.001	**0.049**	-

LDNs and MDSC subsets in subgroups

*Abbreviations:* AUC, area under the curve; CI, confidence interval; PPV, positive predictive value; NPV, negative predictive value; LDNs, low-density neutrophils; MDSCs, myeloid-derived suppressor cells; M-MDSCs, monocytic MDSCs; PMN-MDSCs, polymorphonuclear MDSCs; PCT, procalcitonin; CRP, C-reactive protein

*P* values in bold indicated that the diagnostic value of biomarkers showed significant differences compared to CRP and PCT.

### LDNs and MDSC subsets in subgroups

**Fig. 4:**
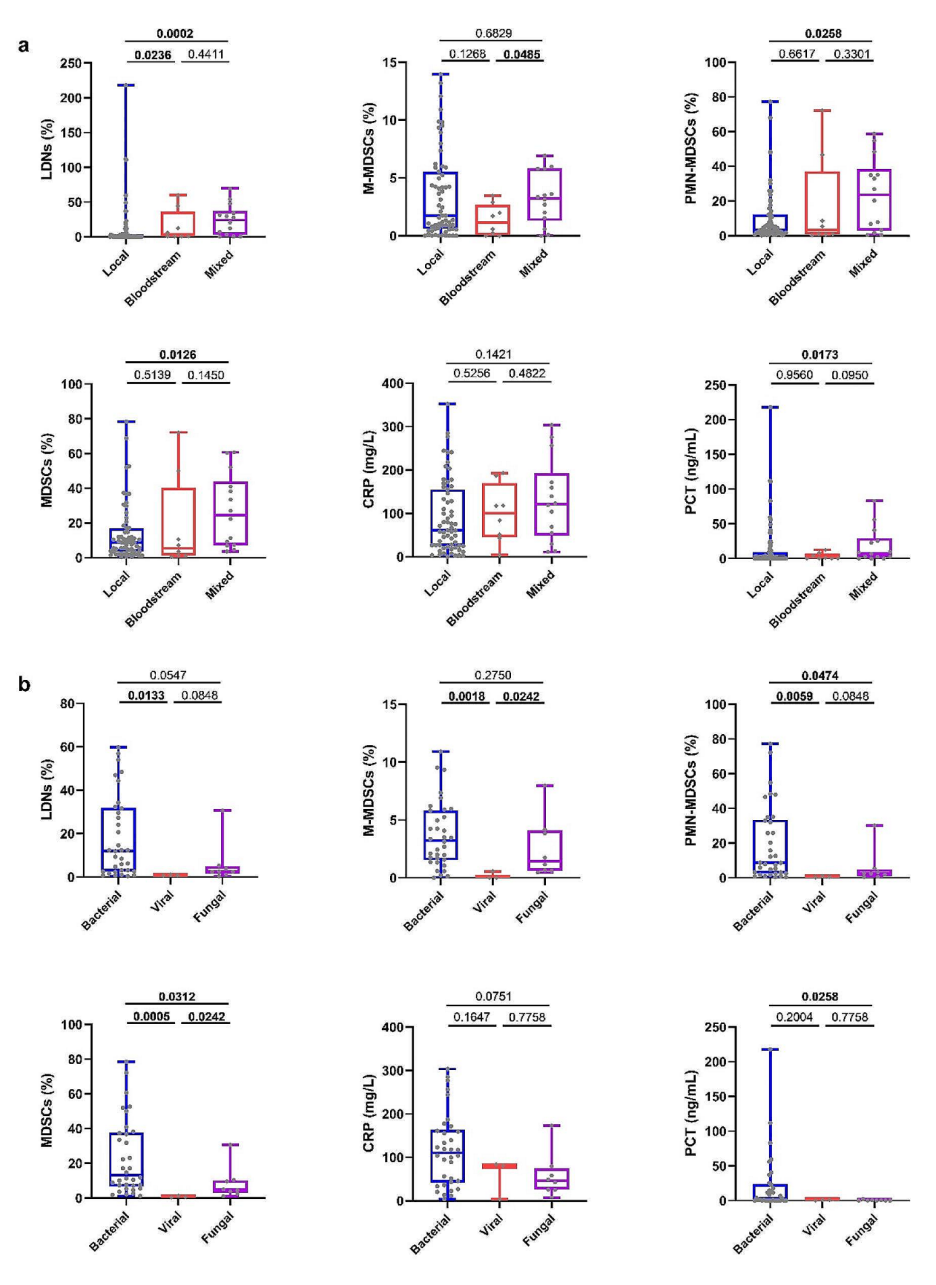
LDNs, MDSC subsets, CRP and PCT in different subgroups: infection site subgroups (**a**), pathogenic pathogens subgroups (**b**). *Abbreviations:* LDNs, low-density neutrophils; MDSCs, myeloid-derived suppressor cells; M-MDSCs, monocytic MDSCs; PMN-MDSCs, polymorphonuclear MDSCs; PCT, procalcitonin; CRP, C-reactive protein

Taking infection sites into account, the characteristics of patients with infection or sepsis were presented in Table S1. LDNs (24.11% vs. 0.28%) and PMN-MDSCs (23.60% vs. 3.29%) were significantly higher in the local-bloodstream mixed infection group than in the local infection group. In addition, the percentage of M-MDSCs was higher in the mixed infection group 3.24 (1.30–5.82)% than in bloodstream infection group 1.13 (0.06–2.68)%. Moreover, LDNs were significantly lower in the local infection group 0.28 (0.09–3.99)% than in the bloodstream infection group 3.49 (0.995–36.37)% (Fig. 4a).

Considering the nature of the infectious pathogens, the characteristics of patients with infection or sepsis were shown in Table S2. patients with viral infections had a significantly lower percentage of LDNs (0.760 vs.12.09%) and MDSC subsets than did patients with bacterial infections. Furthermore, the percentage of M-MDSCs distinguished fungal infections 1.446 (0.623–4.098)% from viral infections 0.002 (0–0.563)%, whereas the percentage of PMN-MDSCs distinguished fungal infections 2.579 (0.962–4.929)% from bacterial infections 8.694 (2.627–33.43)%. MDSCs decreased gradually in bacterial infection 13.31 (6.703%–37.61)%, fungal infection 4.765 (2.878–10.19)%, and viral infections 0.627 (0.382–1.489)% (Fig. 4b).

### LDNs and MDSC subsets were positively associated with sepsis severity

**Fig. 5:**
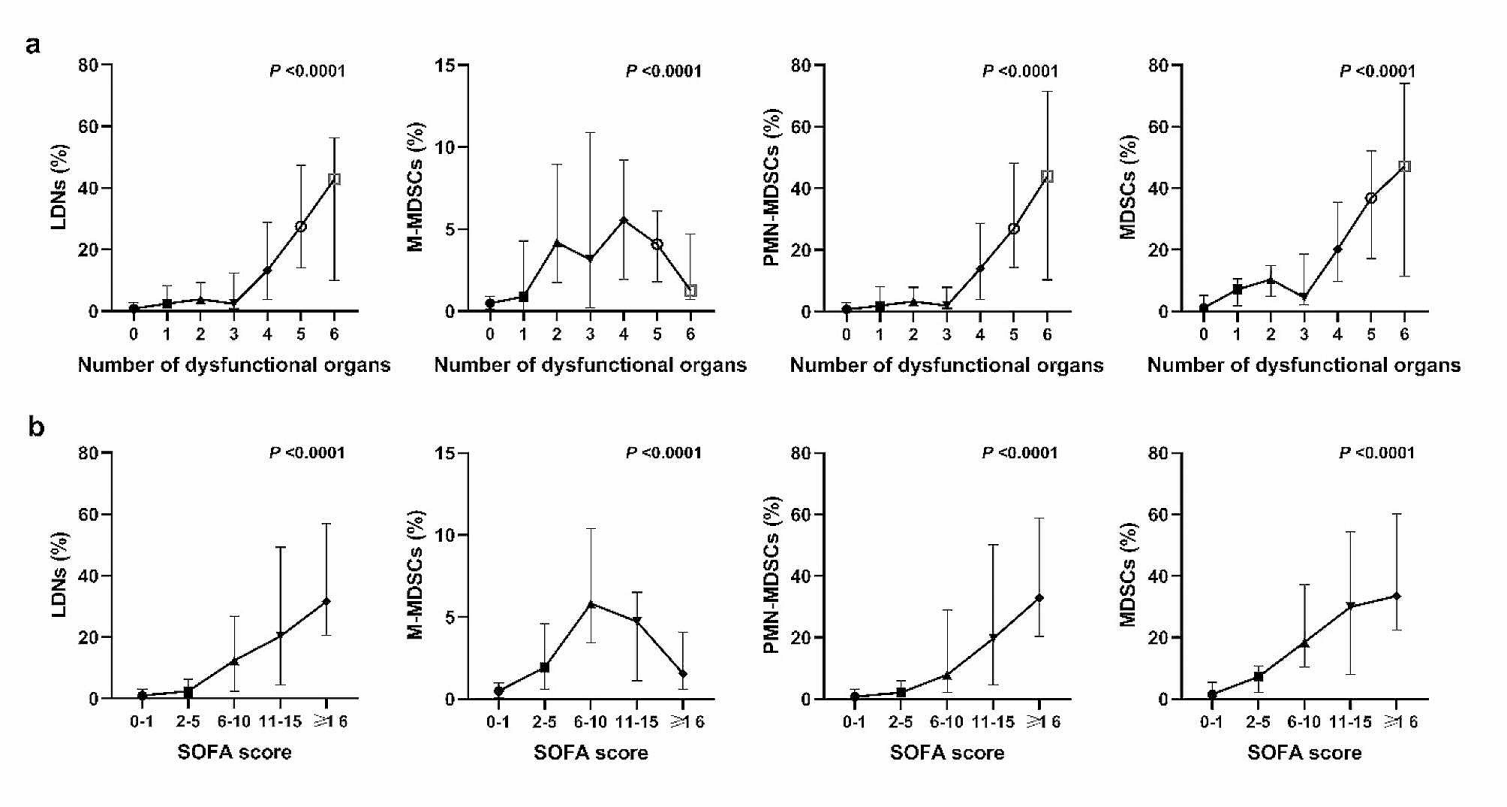
The association of LDNs and MDSC subsets with sepsis severity: with the number of dysfunctional organs (**a**), with SOFA score (**b**). Bar graphs show the median and interquartile range. *Abbreviations:* LDNs, low-density neutrophils; MDSCs, myeloid-derived suppressor cells; M-MDSCs, monocytic MDSCs; PMN-MDSCs, polymorphonuclear MDSCs; SOFA, sequential organ failure assessment

Sepsis severity was measured using SOFA scores and the number of dysfunctional organs. The levels of LDNs and MDSC subsets increased with the number of dysfunctional organs (Fig. 5a). The percentages of LDNs and MDSC subsets were significantly elevated with the SOFA score (Fig. 5b). Additionally, there was 1 (3.03%) secondary infection patient in infection group, whereas there were 23 (44.23%) in sepsis group. The levels of LDNs and MDSC subsets were notably higher in the group with secondary infection (*n* = 24) compared to the group without it (*n* = 61) (Fig. S3). Among the patients with sepsis, 42 did not have septic shock while 10 did. LDNs, PMN-MDSCs, MDSCs and CRP were more abundant in sepsis patients with shock compared to those without (Fig. S4).

### M-MDSCs as a prognostic predictor for the 28-day mortality rate of sepsis

**Fig. 6:**
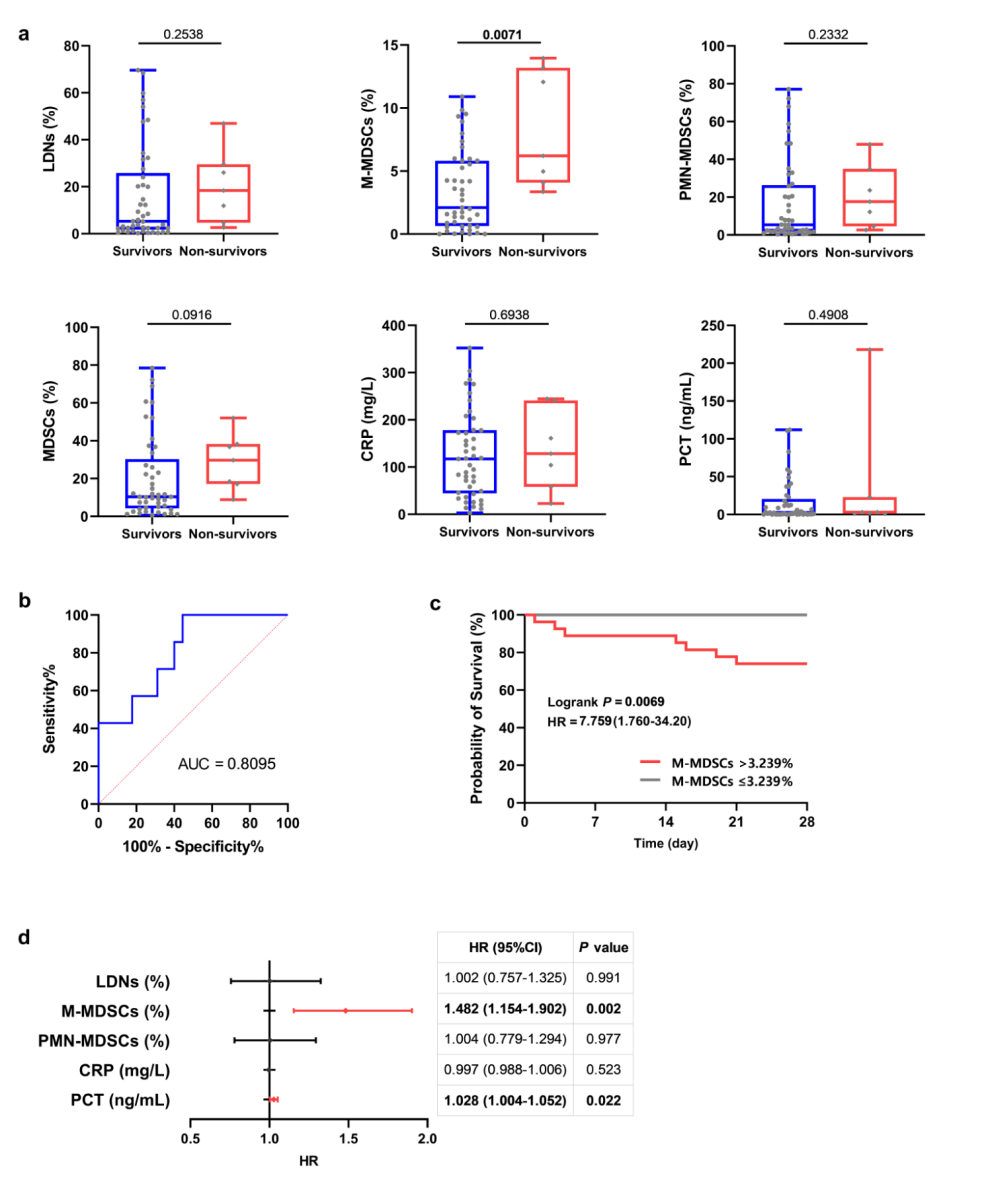
The prognostic value of LDNs and MDSC subsets in the sepsis group. Comparison of LDNs, MDSC subsets, CRP， and PCT between 28-day hospital survivors and non-survivors (**a**). The ROC curve of M-MDSCs for predicting 28-day mortality (**b**). A Kaplan–Meier survival curve was drawn to show the 28-day survival rate for sepsis patients (**c**). Univariate Cox proportional hazards regression analysis of prognostic factors in sepsis patients (**d**). HR and CIs significantly associated with sepsis were shown in red when HR > 1 and in blue when HR < 1. *Abbreviations:* LDNs, low-density neutrophils; MDSCs, myeloid-derived suppressor cells; M-MDSCs, monocytic MDSCs; PMN-MDSCs, polymorphonuclear MDSCs; CRP, C-reactive protein; PCT, procalcitonin; AUC, area under the curve; HR, hazard ratio; CI, confidence interval

There were 45 patients who survived sepsis (38 in non-shock and 7 in shock) and 7 who did not survive (4 in non-shock and 3 in shock) within 28 days of enrollment. Non-survivors 6.201 (4.084–13.20)% had significantly higher percentages of M-MDSCs compared to survivors 2.112 (0.641–5.821)%; however, there were no significant differences in LDNs, PMN-MDSCs, MDSCs, CRP, or PCT levels between survivors and non-survivors (Fig. 6a). The AUC of M-MDSCs for evaluating sepsis prognosis was 0.810 (95% CI 0.656–0.963). The optimal cutoff value was 3.239%, with a sensitivity of 100% and a specificity of 55.56% (Fig. 6b). Sepsis patients with M-MDSCs > 3.239% showed a 7.759 hazard ratio compared to sepsis patients with M-MDSCs ≤ 3.239% in terms of 28-day mortality (Fig. 6c). Furthermore, Cox regression analysis demonstrated that percentages of M-MDSCs were associated with a high 28-day mortality rate in patients with sepsis, as was PCT (Fig. 6d).

## Discussion

An excessive pro-inflammatory response may result in tissue damage and organ failure in patients with sepsis. Concurrently, persistent anti-inflammatory responses promote sustained immunosuppressive environments [17]. Sepsis-induced immunosuppression is a critical factor in sepsis mortality [6]. Providing direct evidence of the severity of the infection and the immune status of patients is vital. PCT and CRP were the most frequently studied biomarkers in patients with infections between 2009 and 2019 [18]. We observed that the LDNs and MDSC subsets were positively correlated with CRP and PCT levels, suggesting that they could also be potential infection biomarkers.

Neutrophils play a protective role in the immune response to invading pathogens [19]. Unlike normal neutrophils, LDNs show obvious abnormal immunological functions, evidenced by myelocyte-like or band-shaped nuclei, immature granulocytes, multilobed nuclei, and mature neutrophils [20]. LDNs were first separated using Ficoll-Hypaque gradients and first identified in PBMCs of patients with SLE, rheumatoid arthritis, and acute rheumatic fever [21]. Our results showed that patients with infection or sepsis had higher LDN levels than healthy controls (HCs). This result is similar to another study in which LDN levels were higher in 26 patients with sepsis admitted to the intensive care unit (ICU) than those in 15 HCs [20].

With regards to infection sites, LDN levels were higher in the local-bloodstream mixed infection group than those in the local infection group. LDNs can distinguish infections in bloodstream from local infections. In our study, LDNs were also elevated in bacterial infections, whereas LDN levels were normal in viral infections. Therefore, LDNs have the potential to accurately distinguish bacterial from viral infections. This is a similar finding to a previous study, in which a large quantity of LDNs was found in the peripheral blood of patients with bacterial infections [22]. LDNs are associated with a higher incidence of secondary infections. In one study, high initial levels of LDNs were associated with a higher risk of secondary nosocomial infections among patients with sepsis [23], likely because LDNs produce high levels of mitochondrial reactive oxygen species (ROS) and neutrophil extracellular traps (NETs) [9, 24, 25]. Spontaneous NETs contain elevated levels of oxidized mitochondrial DNA, which can lead to the synthesis of IFN-β, promote endothelial damage and vasculopathy, activate platelets, and potentially promote thrombosis [26].

M-MDSCs and PMN-MDSCs are pathologically activated neutrophils and monocytes [15]. Therefore, we analyzed MDSC subsets according to their origin. We observed that the monocyte counts were higher in the infection and sepsis groups than in the HCs group, but there was no significant difference between the infection and sepsis groups. The percentage of monocytes was highest in the infection group, followed by the healthy controls, and finally the sepsis group. The neutrophil count and percentage, MLR, and NLR gradually increased in the HCs, infection, and sepsis groups. This supports our finding that mature and immature neutrophil levels were proportionally increased in sepsis compared to those in HCs, whereas monocyte levels were decreased [27].

MDSC-like cells (MDSC-LC) can be screened based on their phenotype. MDSCs-LC with immunosuppressive function can be identified as MDSCs [16, 28]. We did not test and verify the function of MDSCs. Still, our study exhibited M-MDSCs and PMN-MDSCs levels were lowest in the HCs group, elevated in the infection group, and highest in the sepsis group. Mathias et al. have mentioned MDSCs increased rapidly and permanently in patients hospitalized in the ICU for more than 28 days [29]. Furthermore, genes associated with MDSCs recruitment, phenotype, and suppressive functions were upregulated, whereas those associated with adaptive immunity and inflammation were downregulated in a cohort of 29 patients with sepsis and 15 healthy donors [23]. Uhel et al. demonstrated that M-MDSCs were increased in ICU patients with and without sepsis and that CD14^−^CD15^+^ low-density PMN-MDSCs were specifically increased in patients with sepsis [23].

In subgroup analysis of infection sites, among 63 local infections, 8 bloodstream infections, and 14 bloodstream-local mixed infection group, we found M-MDSCs were lower in bloodstream infections than those in mixed infections, whereas PMN-MDSCs were lower in local infections than those in bloodstream-local mixed infections. Analysis of infectious pathogens subgroup showed that M-MDSCs play a key role in the identification of fungal and viral infections, whereas PMN-MDSCs play a major role in the identification of fungal and bacterial infections. In addition, both M-MDSCs and PMN-MDSCs levels were higher in bacterial infections compared to those in viral infections. This result is consistent with that of a study discovered the number of circulating CD14^+^HLA-DR^lo/−^ M-MDSCs was higher in patients with gram-negative sepsis than in those with gram-positive sepsis [23].

The SOFA score is associated with the severity of organ dysfunction and indicates an increased probability of mortality in patients with sepsis [1]. The LDNs and MDSC subsets were positively associated with the SOFA score and the number of dysfunctional organs, suggesting that they could be potential prognostic biomarkers of sepsis. High initial levels of PMN-MDSCs are associated with a higher risk of secondary nosocomial infections among patients with sepsis. A previous study demonstrated that M-MDSCs were not significantly different between 9 patients with nosocomial infection and 27 patients without nosocomial infection [23]; however, we found both PMN-MDSCs and M-MDSCs were associated with a higher incidence of secondary infections. This difference in result could be due to the number of participants in the study. Here, we analyzed 24 and 61 patients with and without secondary infections, respectively. Moreover, our study demonstrated the percentage of M-MDSCs in PBMCs instead of in whole blood. Despite M-MDSCs levels not differing significantly between septic patients with and without shock, MDSCs were higher in the shock subgroup than in the non-shock subgroup. This might be because the number of patients with sepsis was too small to produce statistical evidence.

Patients who died early had the highest initial percentage of MDSCs at 12h and 24 h [29]. For example, in an animal study, T cell proliferation was reduced by 75% in the presence of MDSCs derived from septic mice and by 40% in the presence of MDSCs from uninfected controls, demonstrating that MDSCs from septic neonates were more suppressive [30]. In humans, MDSCs from patients with sepsis significantly suppress the ability of healthy control T cells to produce IFN-γ and IL-4 cytokines [29]. Zhao et al. found that MDSCs secrete itaconate to suppress CD8^+^ T cells proliferation, cytokine production and cytotoxicity [31]. Hollen et al. demonstrated that MDSCs significantly increased in hospitalized sepsis survivors at least 6 weeks after infection. However, MDSCs isolated from sepsis exhibit immunosuppressive function by inhibiting T cells proliferation and IL-2 production only at or after 14 days following sepsis onset [32]. MDSCs not only suppress the number and function of T cells, but also enhance the number of Treg [33], and produce cytokines with immunosuppressive function, such as IL-10 [34] and TGF-β [35], impacting the prognosis of patients with sepsis.

There was no research on the prognosis of sepsis by use of LDNs and MDSCs. The only one report about the prognostic value of M-MDSCs in sepsis was published by Schrijver et al. in 2022. It showed that survivors expressed 1.64-fold more M-MDSCs than 28-day non-survivors in pneumosepsis [36]. In our study, LDNs and MDSCs were positively associated with sepsis severity. Therefore, we evaluated their prognostic value in survivors and non-survivors. M-MDSC levels were higher in non-survivors than in survivors, and Cox regression analysis demonstrated that M-MDSC was an independent predictor for 28-day mortality. This may be one of the factors related to the prognostic value of M-MDSCs in sepsis, when CD14^+^ cells (M-MDSCs) were depleted, CD4^+^ and CD8^+^ T cells in patients with sepsis proliferated rapidly [23].

Our results illustrate that PCT levels did not differ between sepsis survivors and non-survivors. However, Cox regression analysis demonstrated that PCT was associated with a high 28-day mortality rate in patients with sepsis. Through an analysis of 249 patients who were suspected of having sepsis in the emergency department, Lee et al. also reported that PCT was not an independent predictor for mortality [37]. Manifold studies have been published on PCT in sepsis, with some conflicting results on whether PCT can predict prognosis in patients with sepsis. Comparison of 159 survivors and 26 non-survivors of sepsis with suspected bacterial infections showed that PCT could predict poor prognosis of sepsis [38], in agreement with the results of Yang [39] and Mustafic [40]. This difference may be related to the number of study participants and the types of pathogens, even the infection sites causing sepsis.

Our study had some limitations. First, LDNs and MDSCs were isolated using density gradient centrifugation, making it difficult to integrate them into routine clinical practice. Second, we did not continuously monitor LDNs and MDSC subsets to determine their dynamic changes. Furthermore, studies are needed to examine a larger number of samples and patients from another research center to increase the diversity of the patient cohorts.

## Conclusion

Our study showed that LDN and MDSC subsets exhibit good diagnostic performances for infection and sepsis. MDSC was a diagnostic biomarker comparable to CRP and PCT, in infection and sepsis, including for distinguishing sepsis from infection. Notably, M-MSDCs levels were higher in non-survivors than in survivors of sepsis. M-MDSCs showed potential as prognostic biomarker of sepsis and may be useful to predict 28-day hospital mortality in patients with sepsis.

### Electronic supplementary material

Below is the link to the electronic supplementary material.

Supplementary Material 1

## Data Availability

Raw data and materials are available in supplementary files. Further data and materials can be addressed to Cuijuanjuan_sdfyy@163.com. Further inquiries can be directed to Zhaoweifeng@suda.edu.cn.

## Abbreviations

AUC: area under the curve
CI: confidence interval
CRP: C-reactive protein
HR: hazard ratio
HCs: healthy controls
ICU: intensive care unit
IFN: interferon
IL: interleukin
LDNs: low-density neutrophils
MDSCs: myeloid-derived suppressor cells
MLR: monocyte-to-lymphocyte ratio
M-MDSCs: monocytic myeloid-derived suppressor cells
NLR: neutrophil-to-lymphocyte ratio
NPV: negative predictive value
OR: odds ratio
PBMCs: peripheral blood mononuclear cells
PCT: procalcitonin
PMN-MDSCs: polymorphonuclear myeloid-derived suppressor cells
PPV: positive predictive value
ROC: receiver operator characteristics
SOFA: sequential organ failure assessment
TGF: transforming growth factor
