# Supplementary material for: Monocytic myeloid-derived suppressor cells as an immune indicator of early diagnosis and prognosis in patients with sepsis
Source: BMC Infect Dis. 2024 Apr 13;24:399. doi: 10.1186/s12879-024-09290-4 (PMC11015644; doi:10.1186/s12879-024-09290-4)
Supplement: Supplementary file 1 — Supplementary Material 1 [file 12879_2024_9290_MOESM1_ESM.docx]

Supplementary Material

Monocytic myeloid-derived suppressor cells as an immune indicator of early diagnosis and prognosis in patients with sepsis

Juanjuan Cui, MS^1^; Wen Cai, MM^1^; Jing Lin, MD^1^; Li Zhang, BM^2^; Youhan Miao, MD^3^; Ying Xu, MM^1^; Weifeng Zhao, MM^1*^

*** Correspondence:** Weifeng Zhao [Zhaoweifeng@suda.edu.cn](mailto:Zhaoweifeng@suda.edu.cn)


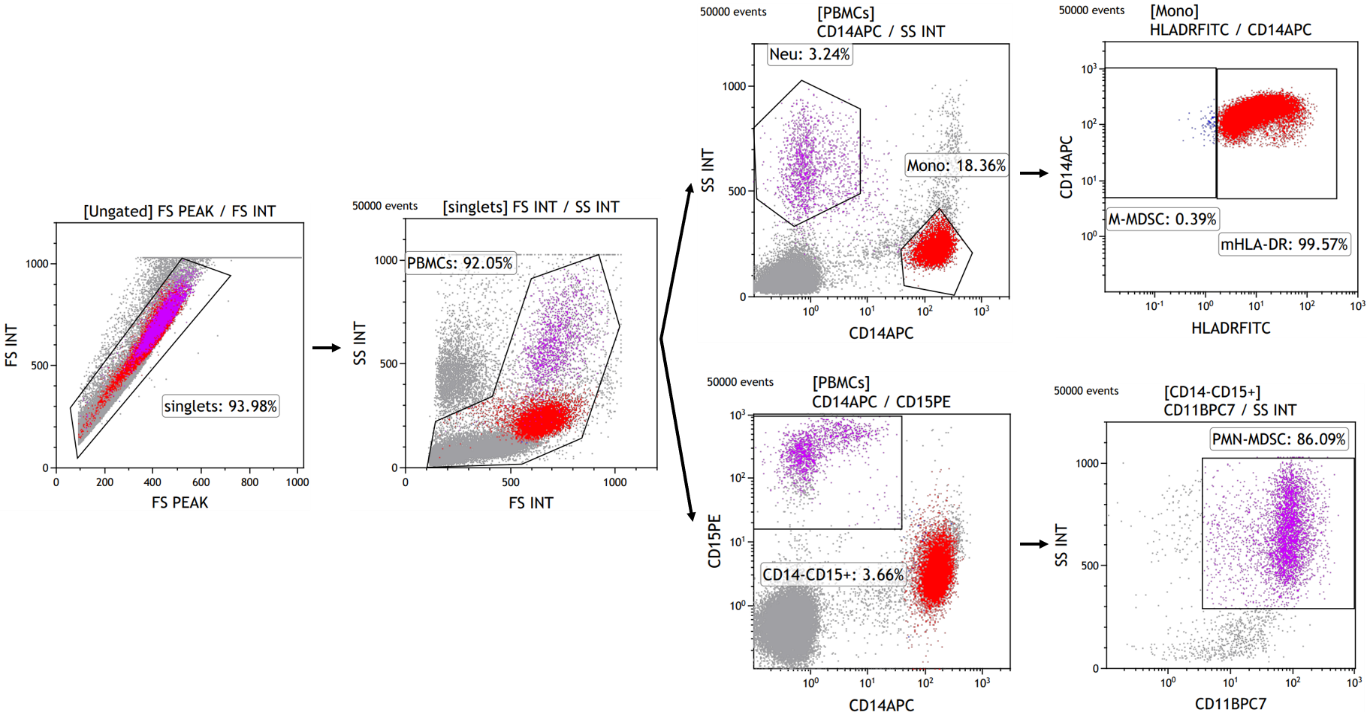


**Fig. S1** Gating strategy for the identification of LDNs, M-MDSCs, and PMN-MDSCs. Cell debris and doublets are excluded. Monocytes and neutrophils are gated based on CD14 and SSC parameters. CD14^-^SS^+^ cells in PBMCs are defined as LDNs. CD14^+^ monocytes are divided into HLA-DR^+^ and HLA-DR^-^ subsets using FMO control. CD14^+^HLA-DR^-/lo^ cells are defined as M-MDSCs. CD14^-^CD15^+^ cells are divided into CD11b^+^ and CD11b^-^ subsets using FMO control. CD14^-^CD15^+^CD11b^+^SS^+^ cells are defined as PMN-MDSCs. Abbreviations: LDNs, low-density neutrophils; M-MDSCs, monocytic myeloid-derived suppressor cells; PMN-MDSCs, polymorphonuclear myeloid-derived suppressor cells.


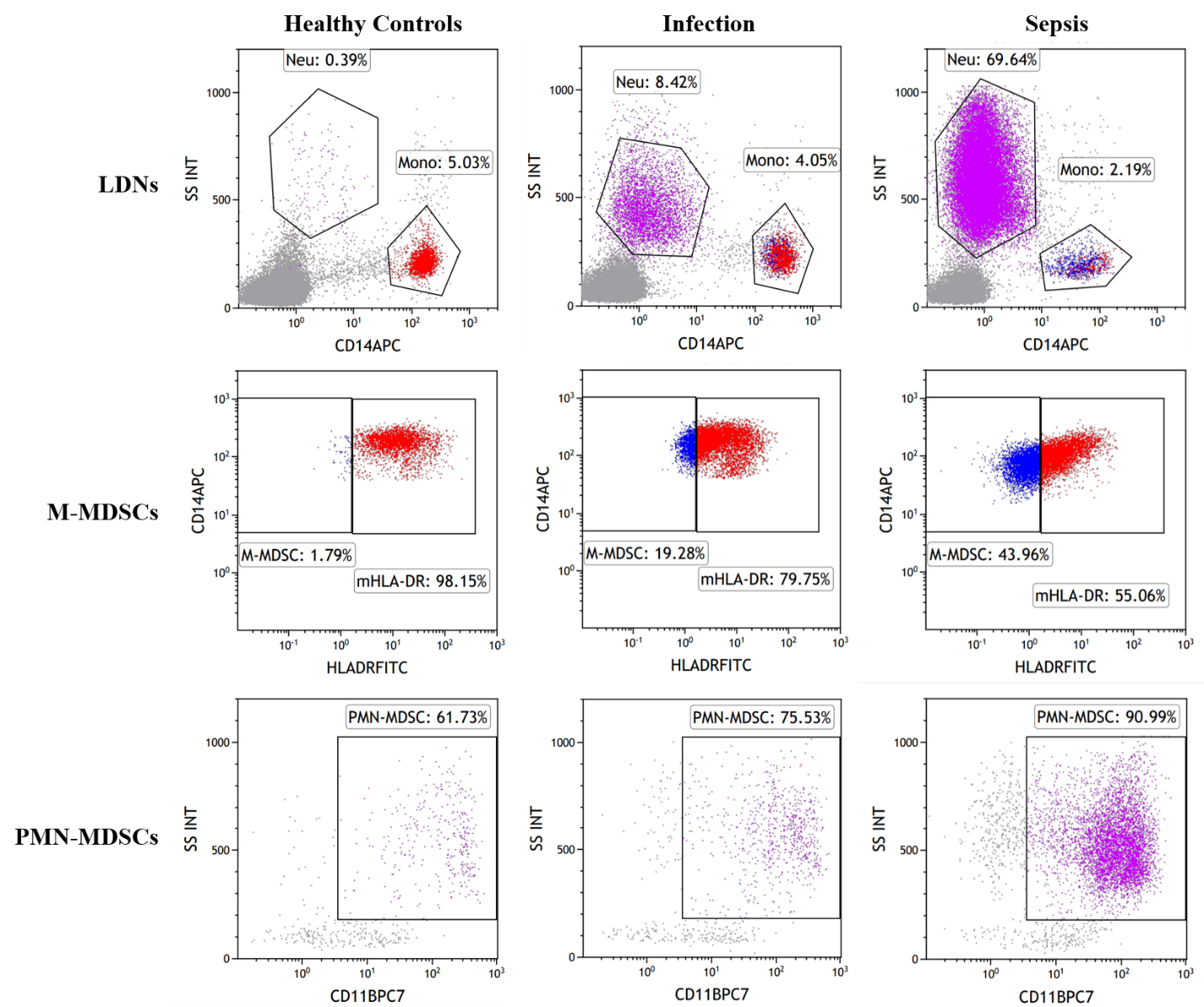


**Fig. S2** Typical flow diagrams of LDNs, M-MDSCs, and PMN-MDSCs in healthy controls, infection, and sepsis groups are shown separately. Abbreviations: LDNs, low-density neutrophils; M-MDSCs, monocytic myeloid-derived suppressor cells; PMN-MDSCs, polymorphonuclear myeloid-derived suppressor cells.

**
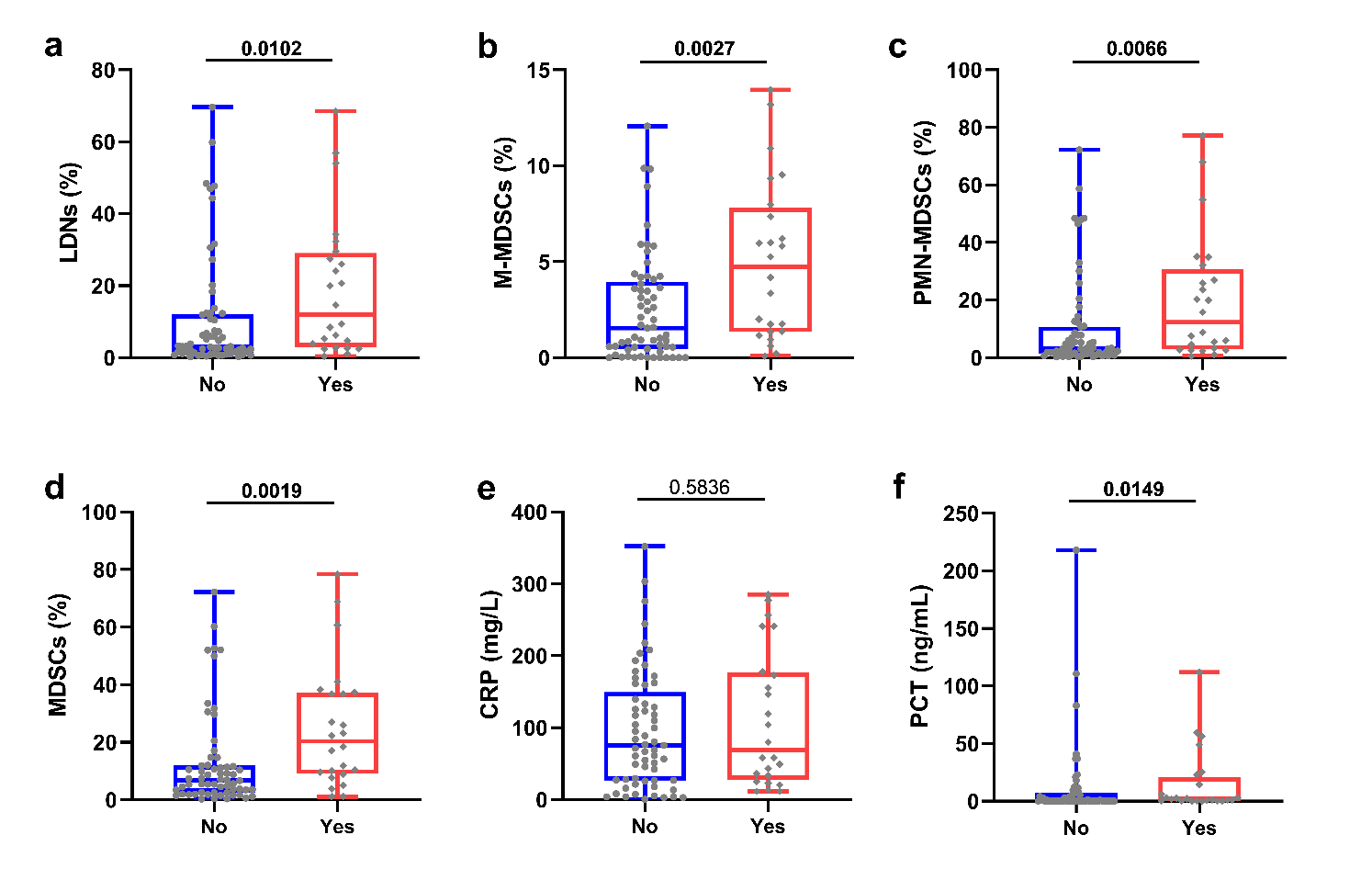
**

**Fig. S3** Comparison of LDNs (a), M-MDSCs (b), PMN-MDSCs (c), MDSCs (d), CRP (e), and PCT (f) between patients with infection or sepsis in secondary infection Yes or No groups. Abbreviations: LDNs, low-density neutrophils; MDSCs, myeloid-derived suppressor cells; M-MDSCs, monocytic MDSCs; PMN-MDSCs, polymorphonuclear MDSCs; CRP, C-reactive protein; PCT, procalcitonin.

**
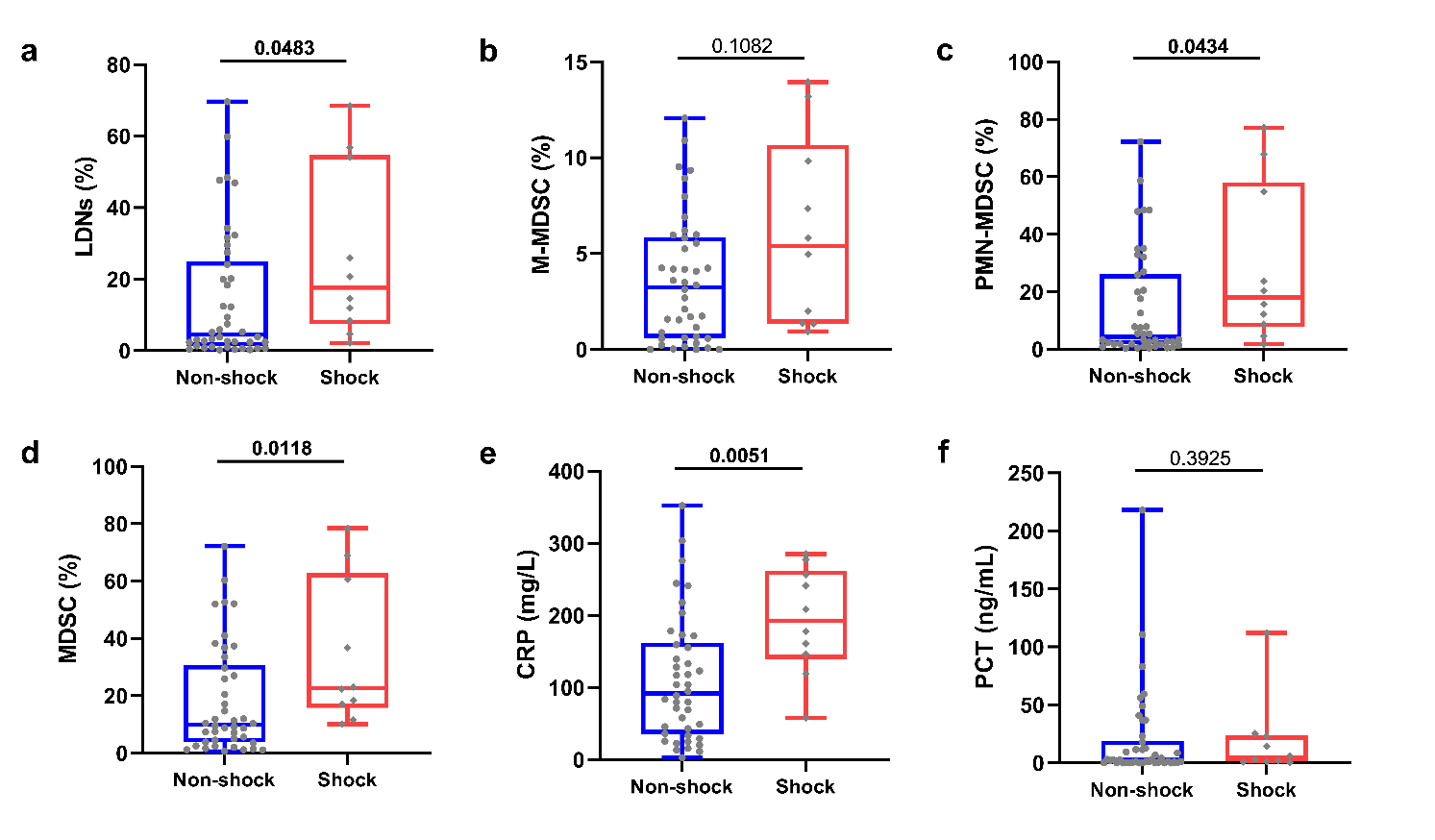
**

**Fig. S4** Comparison of LDNs (a), M-MDSCs (b), PMN-MDSCs (c), MDSCs (d), CRP (e), and PCT (f) between patients with sepsis in the non-shock and shock groups. Abbreviations: LDNs, low-density neutrophils; MDSCs, myeloid-derived suppressor cells; M-MDSCs, monocytic MDSCs; PMN-MDSCs, polymorphonuclear MDSCs; CRP, C-reactive protein; PCT, procalcitonin.

**Table S1** Infection sites in patients with infection and sepsis

|  | **Infection** | **Sepsis** | **Total** |
| --- | --- | --- | --- |
| **Local** | 28 (84.84) | 35 (67.31) | 63 |
| Pulmonary | 13 (46.43) | 16 (45.71) |  |
| Urinary | 2 (7.14) | 2 (5.71) |  |
| Digestive | 7 (25.00) | 12 (34.29) |  |
| Pulmonary and digestive | 0 (0.00) | 1 (2.86) |  |
| Other | 6 (21.43) | 4 (11.43) |  |
| **Bloodstream** | 4 (12.12) | 4 (7.69) | 8 |
| **Local and bloodstream** | 1 (3.03) | 13 (25.00) | 14 |

**Table S2** Infectious pathogens in patients with infection and sepsis

|  | **Infection** | **Sepsis** | **Total** |
| --- | --- | --- | --- |
| **Bacterial** | 7 (21.21) | 27 (51.92) | 34 |
| Gram-positve | 1 (14.29) | 6 (22.22) |  |
| *Staphylococcus aureus* | 0 | 4 |  |
| *Enterococcus faecium* | 0 | 2 |  |
| *Oral Streptococci* | 1 | 0 |  |
| Gram-negative | 6 (85.71) | 17 (62.96) |  |
| *Klebsiella pneumoniae* | 3 | 9 |  |
| *Acinetobacter baumannii* | 1 | 2 |  |
| *Escherichia coli* | 0 | 3 |  |
| *Haemophilus influenzae* | 0 | 1 |  |
| *Enterobacter aerogen* | 0 | 1 |  |
| *Proteus mirabilis* | 0 | 1 |  |
| *Pseudomonas aeruginosa* | 1 | 0 |  |
| *Salmonella enterica* | 1 | 0 |  |
| Gram-positive and -negative | 0 (0.00) | 4 (14.81) |  |
| **Viral** (Epstein-Barr virus) | 1 (3.03) | 2 (3.85) | 3 |
| **Fungal** (*Candida albicans*) | 2 (6.06) | 6 (11.54) | 8 |
| Other | 19 (57.58) | 10 (19.23) | 29 |
| Undetectable | 4 (12.12) | 7 (13.46) | 11 |
